# Objective and subjective assessment of sleep in chronic low back pain patients compared with healthy age and gender matched controls: a pilot study

**DOI:** 10.1186/1471-2474-10-122

**Published:** 2009-10-02

**Authors:** Grainne M O'Donoghue, Niall Fox, Conor Heneghan, Deirdre A Hurley

**Affiliations:** 1 School of Physiotherapy and Performance Science, Health Sciences, University College Dublin, Belfield, Dublin 4, Ireland; 2 BiancaMed, Nova UCD, Belfield, Dublin 4, Ireland; 3 School of Electrical, Electronic and Mechanical Engineering, University College Dublin, Belfield, Dublin 4, Ireland

## Abstract

**Background:**

While approximately 70% of chronic low back pain (CLBP) sufferers complain of sleep disturbance, current literature is based on self report measures which can be prone to bias and no objective data of sleep quality, based exclusively on CLBP are available. In accordance with the recommendations of The American Sleep Academy, when measuring sleep, both subjective and objective assessments should be considered as the two are only modestly correlated, suggesting that each modality assesses different aspects of an individual's sleep experience. Therefore, the purpose of this study was to expand previous research into sleep disturbance in CLBP by comparing objective and subjective sleep quality in participants with CLBP and healthy age and gender matched controls, to identify correlates of poor sleep and to test logistics and gather information prior to a larger study.

**Methods:**

15 CLBP participants (mean age = 43.8 years (SD = 11.5), 53% female) and 15 healthy controls (mean age = 41.5 years (SD = 10.6), 53% female) consented. All participants completed the Pittsburgh Sleep Quality Index, Insomnia Severity Index, Pittsburgh Sleep Diary and the SF36v2. CLBP participants also completed the Oswestry Disability Index. Sleep patterns were assessed over three consecutive nights using actigraphy. Total sleep time (TST), sleep efficiency (SE), sleep latency onset (SL) and number of awakenings after sleep onset (WASO) were derived. Statistical analysis was conducted using unrelated t-tests and Pearson's product moment correlation co-efficients.

**Results:**

CLBP participants demonstrated significantly poorer overall sleep both objectively and subjectively. They demonstrated lower actigraphic SE (p = .002) and increased WASO (p = .027) but no significant differences were found in TST (p = .43) or SL (p = .97). Subjectively, they reported increased insomnia (p =< .001), lower SE (p =< .001) and increased SL (p =< .001) but no difference between TST (p = .827) and WASO (p = .055). Statistically significant associations were found between low back pain (p = .021, r = -.589), physical health (p = .003, r = -.713), disability levels (p = .025, r = .576), and subjective sleep quality in the CLBP participants but not with actigraphy.

**Conclusion:**

CLBP participants demonstrated poorer overall sleep, increased insomnia symptoms and less efficient sleep. Further investigation using a larger sample size and a longer period of sleep monitoring is ongoing.

## Background

Chronic low back pain (CLBP) is among the most prevalent of all health complaints, is associated with enormous health care costs, reduced quality of life, and is almost universally associated with insomnia [1]. Two recent studies reported that 55% of chronic low back pain (CLBP) sufferers subjectively report impaired sleep after pain onset [2], and over half of this population suffers from moderate or severe co-morbid insomnia [3]. A study in France investigating subjective sleep quality in 101 CLBP patients found that their sleep was significantly altered compared to that of healthy age, sex and height matched controls [4].

The majority of previous research into sleep disturbance and musculoskeletal pain has been based upon self-report measures in heterogeneous patient groups (e.g. headache, neck pain, back pain, fibromyalgia) [5-8]. The main difficulty in interpreting these results is the variety of populations studied as individuals that present to an orthopaedic out-patient clinic with back pain are likely different from in-patients with chronic headaches. Therefore, further studies of homogenous patient populations are needed to form generalisations about sleep and pain and specific clinical conditions [9].

The criterion standard for measuring sleep is polysomnography (PSG) [10]: an objective measurement that assesses actual physical and physiological processes during sleep-activity periods. However, because of its inherent logistical complexity and expense, studies that document sleep disturbance by PSG are infrequent in the literature and have mostly concentrated on small samples of patients with fibromyalgia [10]. Its use has been limited to one study in sleep disturbance and CLBP, which found CLBP participants reported significant levels of sleep disturbance as compared to controls, but all groups had equivalent amounts of sleep and comparable sleep architecture on PSG [11]. However, this study controlled for many factors other than pain including depression that may contribute to the sleep complaints in this population. The authors consequently reported that the absence of signs of major sleep disturbance must not be interpreted as evidence of a lack of a true sleep problem in CLBP but more likely reflects control of these factors, as well as the difficulty in measuring sleep. Additionally, PSG generally requires that patients do not take CNS active medications for two weeks prior to assessment. This requirement is a stringent one and can be a limitation for many individuals treated for chronic pain conditions, including low back pain [10].

An alternate, less expensive and less obtrusive objective method for detecting sleep is wrist actigraphy [10]. The actigraph distinguishes between sleep and wakefulness using activity counts as a proxy measure of sleep [12] and is particularly useful as it does not require participants to spend the night in an artificial sleep laboratory environment and allows for consecutive nights monitoring. It has demonstrated reasonably high epoch by epoch (> 0.85) and sleep efficiency agreement with PSG (> 0.80) [12].

Nonetheless, only one study in a mixed group of chronic pain participants (including CLBP) has measured sleep using both actigraphy and self report measures finding that pain severity was associated with subjective sleep disturbance but not actigraphy, for individuals reporting high pain severity [8]. The American Sleep Academy recommends that both subjective and objective measures of sleep are warranted in musculoskeletal pain research, as the two are only modestly correlated [6,7] and they assess different aspects of an individual's sleep experience [13,14].

The purpose of this pilot study was to expand previous research into sleep disturbance in CLBP by conducting both subjective and objective assessments and to test logistics and gather information prior to a larger study, in order to improve the latter's quality and efficiency.

We hypothesised that CLBP patients would demonstrate on actigraphy reduced total sleep time (TST), increased sleep onset latency (SL), increased waking after sleep onset (WASO) and decreased sleep efficiency (SE), and on subjective measures, poorer overall sleep quality and higher insomnia scores, compared to healthy age and gender matched controls. It was suggested that there would be a moderate to strong association between pain, quality of life, disability and sleep quality in CLBP participants and that moderate associations would be found between subjective and objective sleep measures.

## Methods

A cross sectional study involving 15 control participants and 15 CLBP participants utilising objective and self report methods of measuring sleep quality in free living conditions was conducted.

### Participants

The control group was recruited via poster advertisement in a university campus. Posters which described the nature of the study and provided a contact number were placed throughout the campus. Following initial contact by the volunteers, telephone screening for eligibility was conducted. Volunteers were recruited if they were aged between 18 and 65 years, were pain-free, currently of good health and reported no diagnosed sleep disturbances.

The CLBP participants were recruited from a physiotherapy waiting list in a large city hospital. Twenty eight potential participants were contacted by letter and invited to take part in the study. Participants were included if they had a history of low back pain of more than 12 weeks duration and excluded if they had any serious spinal pathology, major active psychotic illness and/or a primary diagnosis of insomnia.

All potential participants were invited to meet the researcher and received written and verbal information about the study. An appointment was made for one week later for each subject to provide informed consent, participate in a baseline assessment and receive the study instructions. Ethical approval was obtained from the University Ethics Committee and the participating hospitals Medical Ethics Committee.

### Procedures

After initial screening and enrolment, study participants completed a baseline assessment which took approximately 45 minutes. Gender, age, weight, height, BMI and medication usage were also recorded. All participants completed self report measures of sleep quality (Pittsburgh Sleep Quality Index [15], Insomnia Severity Index [16], quality of life (SF36v2) [17] and pain (bodily pain scale of the SF36v2) [17], and for the CLBP participants functional disability, measured by the Oswestry Disability Index (ODI) [18]. All participants then underwent three consecutive nights of actigraphy monitoring, while simultaneously completing a daily sleep log; the Pittsburgh Sleep Diary [19].

### Outcome Measures

#### Objective Sleep Assessment - Actigraphy

Sleep patterns were objectively assessed using the Actiwatch (registered trademark of Mini Mitter Company, Cambridge Neurotechnology Ltd. UK): a compact, light weight, wrist worn electronic device that measures and records physical activity. The Actiwatch contains a sensor capable of detecting acceleration in two planes. It is sensitive to 0.01 g and integrates the degree and speed of motion and produces an electrical current with varying magnitude. An increased degree of speed and motion produces an increase in voltage. The watch converts this signal and stores it as activity counts. The maximum sampling rate is 32 Hz. Sleep variables are extracted from this activity data using the Actiwatch Activity & Sleep Analysis 5 [20].

The watch was worn by the participating participants on their non-dominant wrist. The participants were instructed to press a button on the watch to activate an individual event marker on four occasions: Bed Time, Get Up Time, Sleep Start and Sleep End. The sleep log (PSD) completed by each subject was used to verify and validate the markers on the actigraphy data. The watch was set to record the number of activity counts during 15 second intervals (epochs).

Sleep variables included total sleep time (TST), awakenings after sleep onset (WASO), sleep onset latency (SL) and sleep efficiency (SE). TST represented the total amount of sleep in minutes received from onset of sleep to onset of awakening. WASO referred to the time (minutes) spent awake after sleep onset had occurred. SL was the time period measured from "lights out" or "bedtime" to the beginning of sleep, and SE defined as the proportion of sleep in the period potentially filled by sleep: ratio of total sleep time to time in bed as a percentage, where a SE less than 85% is considered to be indicative of sleep disturbance [21].

#### Self report assessment

##### Pittsburgh Sleep Quality Index

The Pittsburgh Sleep Quality Index (PSQI) measured retrospective sleep quality and disturbances [15]. The PSQI discriminates between good and poor sleepers and provides a brief, clinically useful assessment of multiple sleep disturbances. It comprises of 19 items that generate seven component scores. The sum of these scores (range 0 - 21) yield a global measure of sleep quality, with higher scores indicating increasingly poor sleep (> 5 indicative of sleep disturbance). The components assess a broad range of domains associated with sleep quality including duration of sleep, sleep latency, the frequency and severity of specific sleep related problems and the perceived impact of poor sleep on daytime functioning. This questionnaire is perhaps the most widely used general measure of sleep available and the strengths of this instrument are its range of coverage of multiple dimensions of sleep quality, its flexibility as a brief clinical tool and its demonstrated validity and utility in chronic pain research [22].

##### The Insomnia Severity Index

The Insomnia Severity Index (ISI) measures insomnia severity [16]. While not specifically developed for pain patients, scale development included a heterogenous group of patients with insomnia secondary to pain conditions [23]. It comprises seven items assessing insomnia severity, generating a total that ranges from 0-28 (with higher scores indicative of more severe insomnia).

Following the recommended score interpretation guidelines, a total score of 0-7 indicates 'no clinically significant insomnia', 8-14 'sub-threshold insomnia', 15-21 'clinical insomnia (moderate severity)' and 22-28 'clinical insomnia (severe)' [15]. The cut off level of 14 has optimal sensitivity (94%) and specificity (94%) in distinguishing a group of adults diagnosed with primary insomnia from those without [24].

##### Pittsburgh Sleep diary

The Pittsburgh Sleep Diary (PSD) was used to quantify subjectively reported sleep and wake behaviours [19]. It is divided into two daily questionnaires completed at 'bedtime' and 'waketime'. The timing and duration of various daytime and sleep-wake parameters and activities are hand entered by the participant.

The bedtime component comprises of six general items; (i) the timing of meals, (ii) consumption of caffeine, (iii) alcohol and (iv) tobacco products, (v) medication use and (vi) and the timing and duration of exercise and nap periods. The daytime component gathers data on (i) bedtime, (ii) 'lights out time', (iii) sleep latency, (iv) final waketime, (v) method of final awakening, (vi) frequency of nightly awakenings, (vii) wake after sleep onset time, (viii) reasons for nightly awakenings, (ix) sleep quality and (x) mood on final wakening and (xi) alertness on final wakening.

In addition to categorical and frequency data generated by the night time questionnaire, the wake-time questionnaire permits the calculation of standard continuity parameters as follows:

(i) sleep latency (SL), minutes; ranges from 0 to total time in bed (TIB); (ii) frequency of nightly awakenings; ranges from 0 to 5+; (iii) total sleep time (TST), calculated by the formula: (TST = (TIB - SL + WASO)); ranges from 0 to TIB; (v) sleep efficiency percentage (SE), calculated by the formula, SE = TST/TIB.

The PSD was developed from three investigations including samples of participants with sleep disorders, healthy adult controls, chronic pain patients and older adults. Significant long term test-retest correlation coefficients for the wake-time variables with a mean inter-test interval of 22 months (range; r = 0.59 - 0.81) have been reported [25].

There are no formally established research diagnostic criteria for sleep diary estimates of sleep continuity. Commonly used clinical and research criteria for symptom severity are as follows: mean sleep latency and/or wake after sleep onset time > 30 minutes and mean sleep efficiency percentage < 85% [26].

#### Assessment of Quality of Life and Pain

##### SF-Item Short-Form 36 Health Survey (standard) Version 2.0 (SF36v2)

The SF-36 (36-Item Short-Form Health Survey) was used to measure overall health status, functional status and health-related quality of life [17]. Two standardised summary scores are calculated from eight sub-scores; the Physical Component Score (PCS) and the Mental Component Score (MCS). The bodily pain scale of the SF36v2 was used to measure pain severity. 'How much bodily pain have you had during the past four weeks?' There are six possible answers varying from none to very severe. [17].

#### Assessment of Low Back Pain-related Disability

##### Oswestry Disability Index

The Oswestry Disability Index (ODI) is a self administered valid and reliable questionnaire used in the field of spinal research to indicate the extent to which a person's activities of daily living are disrupted or restricted by low back pain [18]. It consists of ten items and is completed in reference to the patient's functional status 'today'. Each item is scored 0-5; the total score is multiplied by two and expressed by a percentage, with the higher the score, the higher the level of disability.

### Statistical Analysis

Analytic data files were created using SPSS software and all analyses were conducted using SPSS V12.0 for Windows (SPSS Inc., Chicago, IL). Descriptive statistics were computed for demographic and health characteristics and each sleep variable and summarised using mean values. Group differences on categorical variables (physical activity, smoking, caffeine consumption and medication) were evaluated using x^2 ^analysis.

Preliminary assumption testing was conducted to check normality, linearity, univariate and multivariate outliers, homogeneity of variance-covariance matrices and muticollinearity, with no serious violations noted. Unrelated t-tests were used to compare mean values for both objective and subjective sleep parameter scores between CLBP participants and healthy controls. Effect sizes (eta squared) were interpreted using Cohen's guidelines with > 0.2 being a small effect size, > 0.5 a medium effect size and > 0.8 a large effect size.

To assess the association between subjective and objective sleep measures, and to determine if there was any relationship between sleep, pain, quality of life and/or disability, Pearson's product moment correlation co-efficients were performed.

## Results

### Demographics and Health Characteristics

In total, 31 healthy individuals volunteered for the study. The 15 healthy participants (n = 8 female) aged between 24 and 60 years (38.46 ± 10.57) chosen to participate were those closest to the CLBP participants in gender and age. Out of 28 CLBP participants invited to participate, 15 (n = 8 female) aged between 26 and 64 years (45.0 ± 11.53) were recruited. Sample demographics and health characteristics are shown in Table 1. Among the CLBP participants, 86% (n = 13) reported using prescription medication for pain management, and 26% (n = 4) used antidepressants compared to none of the control group (p = .032). In both groups, 26% (n = 4) were medicated to control high blood pressure and/or high cholesterol. Two thirds of the control group (n = 10) reported participating in regular exercise, whereas all of the CLBP participants exercised but to a significantly lesser intensity than the healthy controls (p = .007). Significantly more CLBP participants smoked cigarettes (p = .032), and both groups consumed equal amount of caffeine (≥ one cup per day). Analyses indicated no significant differences between groups in age (p = .117), gender (p = 1.0), weight (p = .29). However, compared to the healthy controls, CLBP participants reported significantly poorer quality of life on both subscales of the SF36 (p =< .001) and more severe pain (p =< .001).

**Table 1:** Demographics and Clinical Characteristics of Sample by Study Group

	**Control Group** **(n = 15)**	**CLBP Group** **(n = 15)**	**t-value**	**p-value**	**CI (95%)**
**Demographics**					
**Age (yrs)** **mean (SD)**	38.5 (10.57)	45.0 (11.53)	-1.617	.117	-1481 - 1.74
**Gender** **% (n) female**	53.3 (n = 8)	53.3 (n = 8)		1.00	
**Weight** **mean (SD)**	74.0 (10.69)	80.7 (21.12)	- 1.090	.285	-19.39 - 6.1
**Physical Activity % (n)**				.007†	
**None**	33.3 (5)	00.0 (0)			
**Low**	20.0 (3)	46.7 (7)			
**Moderate**	13.3 (2)	46.7 (7)			
**High**	33.3 (5)	06.6 (1)			
**Smoker** **% (n)**	0 (0)	26.6 (4)		.032†	
**Caffeine** **% (n)** **(> 1 cup per day)**	86.6 (13)	86.6 (13)		1.00	
**Medication** **% (n)**					
**Pain**	0 (0)	86.6 (13)		<.001†	
**Sleep**	0 (0)	0 (0)		1.00	
**Antidepressants**	0 (0)	26.6 (4)		.032†	
**Others**	26.6 (4)	26.6 (4)		1.00	
**SF 36** **mean (SD)**					
**MCS**	57.3 (3.3)	44.6 (10.1)	4.64	<.001†	6.94 - 18.52
**PCS**	56.4 (3.1)	44.0 (6.3)	6.93	<.001†	8.75 - 16.12
**Bodily Pain**	61.8 (.87)	39.9 (8.1)	10.35	<.001†	17.36 -26.42

† = p < 0.05 MCS: Mental Combined Score	PCS: Physical Combined Score

### Sleep Assessment

The following parameters were derived both objectively from the computer-scored actigraph data and subjectively from the Pittsburgh sleep diary: TST, SE, SL and WASO. Additionally, global sleep quality and insomnia severity were derived from the PSQI and ISI questionnaires respectively.

### Objective Sleep Assessment

Table 2 presents the mean values, standard deviations and confidence intervals for each actigraphic sleep variable by study group. Unrelated t-tests found a significant difference between CLBP participants and healthy controls in SE (t = 3.45, p = .002) and WASO (t = -2.33, p = .027). No significant differences were found in TST (t = .805, p = .428) or SL (t = .036, p = .97). Actigraphy recordings demonstrated that both groups took approximately nine and a half minutes to fall asleep after lights out (SL) and slept for similar amounts of time i.e. approximately six hours 50 mins of estimated sleep per night (TST).

**Table 2:** Differences between mean scores on actigraphic sleep variables for healthy controls and CLBP subjects

	**Control Group**	**CLBP Group**	**t-value**	**p-value**	**CI (95%)**
**Variable**					
**Total sleep time (mins) mean (SD)**	399.13 (40.8)	381.66 (73.5)	.805	.428	-27.01-61.93
**Sleep efficiency (%) mean (SD)**	85.8 (4.4)	77.8 (7.8)	3.45	.002†	3.25 - 12.84
**Sleep onset Latency (mins) mean (SD)**	9.43 (10.2)	9.28 (11.1)	.036	.972	-8.11 - 8.4
**Awakenings after sleep onset (mins) mean (SD)**	7.4 (8.06)	24.4 (30.39)	-2.094	.055	-34.22-.2162

### Subjective Sleep Assessment

Table 3 presents the subjective data. There was a statistically significant difference in self-report sleep onset latency (t = -4.19, p =< .001) and sleep efficiency (t = 4.85, p =< .001) Mean scores indicated that CLBP participants reported significantly lower sleep efficiency scores (mean (SD) = 73.4% (16.5)) than the healthy controls (mean (SD) = 95.3%, (5.8)), and significantly higher sleep onset latency times, up to three times longer (CLBP; mean = 45.3 mins, (27.7)) and controls; mean = 11.73 minutes (14.2)). There was no difference between groups in total sleep time (t = .220, p = .827) or number of minutes awake after sleep onset (t = -2.094, p = .053).

**Table 3:** Differences between mean scores (SD) on subjective sleep variables for healthy controls and CLBP subjects

	**Control Group**	**CLBP Group**	**t-value**	**p-value**	**CI (95%)**
**Variable**					
**Total sleep time (mins) mean (SD)**	439.33 (42.6)	434.33(76.9)	.220	.827	-41.5 - 51.52
**Sleep efficiency (%) mean (SD)**	95.3 (5.8)	73.4 (16.5)	4.849	<.001	12.39 - 31.41
**Sleep onset latency (mins) mean (SD)**	11.7 (4.3)	45.3 (27.7)	-4.185	<.001	-50.3 - -16.89
**Awakenings after sleep onset (mins) mean (SD)**	7.4 (8.06)	24.4 (30.39)	-2.094	.053	-34.22- .2162

Global sleep quality, as measured by the PSQI was reported to be significantly poorer in the CLBP group compared to the control group. In total, 86.6% (n = 13) of the CLBP group scored > 5 on the PSQI, indicative of poor sleep quality, compared to only 6.6% (n = 1) of the control group (p = <0.001). Over half of the CLBP participants (n = 8) reported threshold clinical insomnia (> 13 on ISI) compared to none of the healthy adults (p =< .001) and all CLBP participants reported multiple night time awakenings (mean (SD) = 2.6(1.5) wakening bouts).

### Association between Subjective and Objective Sleep Variables

Significant associations were found between self report and actigraphic total sleep time in the control (r = .78, p = .01) and the CLBP groups (r = .80, p = <0.001; Table 4). Subjective and actigraphically measured sleep latency were significantly correlated in the control group (r = .73, p = .002), but only a small non-significant association was found in the CLBP group (r = .34, p = .25). There was no correlation between subjective and objectively measured WASO in either group (control: r = .006, p = .99, CLBP: r = .072, p = .80). There was a moderate association between self reported and actigraphic sleep efficiency in the control group (r = .42, p = .12), with better subjective sleep quality associated with higher sleep efficiency scores. However, no relationship was found between subjective and objective sleep efficiency in the CLBP participants (r = .28, p = .30).

**Table 4:** Relationship between objectively and subjectively measured sleep variables

	**Objective**	**Subjective**	**r-value**	**p-value**
**Control Group (n-15)**				
**Total Sleep Time (mins) mean (SD)**	399.13 (40.8)	439.33 (42.6)	0.778	<.001 †
**Sleep Efficiency (%) mean (SD)**	85.83 (4.4)	95.33 (5.8)	0.417	.42
**Sleep onset Latency (mins) mean (SD)**	9.43 (10.2)	11.73 (14.2)	0.730	<.002 †
**Awakenings after Sleep Onset (mins) mean (SD)**	32.0 (9.22)	7.4 (8.06)	.006	.984
**CLBP Group (n = 15)**				
**Total Sleep Time (mins) mean (SD)**	434.33 (76.9)	381.66 (73.5)	0.795	<.000†
**Sleep Efficiency (%) mean (SD)**	73.43 (16.5)	77.81 (7.8)	0.298	.33
**Sleep onset Latency (mins) mean (SD)**	45.33 (27.7)	9.28 (11.1)	0.343	.251
**Awakenings after Sleep Onset (mins) mean (SD)**	43.3 (15.8)	24.4 (30.4)	.072	.798

† p < 0.000

### Relationship between Pain, Quality of Life, Disability and Sleep Measures

In the control group, only the Mental Combined Score (MCS) of the SF36v2 was associated with sleep. There was a strong association between the MCS and actigraphy total sleep time (r = .61, p = .017), and a weaker association with sleep efficiency (r = -.49, p = 0.056). Neither pain nor the Physical Component Score of the SF36v2 were significantly associated with any sleep variables in this group.

In the CLBP group, there was a strong relationship between pain and objectively measured sleep but no relationship with quality of life or disability. Strong statistically significant relationships were found between the self report PSQI global score and pain (r = -.59, p = .021), the physical component score of the SF36v2 (r = -.71, p = .003), and functional disability (r = .58, p = .025). Similar relationships were found between the ISI score and pain (r = -.74, p = .001), the physical component score of the SF36v2 (r = -.57, p = .026), functional disability (r = .64, p = .010) and sleep efficiency derived from the PSD (r = -.52, p = .05). There was also a strong statistically significant relationship between both objectively (r = .62, p = .014) and subjectively (r = .54, p = .039) measured sleep efficiency and the physical combined score of the SF36v2, with a higher PCS correlating with better sleep efficiency.

## Discussion

This study has confirmed that CLBP participants subjectively report higher levels of sleep disturbance compared to age and gender-matched controls, and for the first time has compared self report to objective measurement finding a significantly higher level of subjective than objective levels of sleep disturbance in this population. Although small, the sample was representative of the majority of CLBP patients managed at out-patients clinics, in that there was a wide variation in age and duration of symptoms, a higher percentage were women and the majority were taking some form of prescription analgesia at the time of initial evaluation [27].

CLBP participants were found to have poorer overall sleep quality, with higher levels of sleep disturbance and insomnia on subjective measures and lower overall sleep quality on actigraphy compared with healthy age and gender matched controls. The high prevalence of self reported perceived sleep disturbance in CLBP patients found in this study supports previous research in chronic pain populations using subjective measures [5-8,28,29]. Furthermore, the objective measurement of sleep disturbance supplements the evidence base, finding 87% of CLBP participants reported poor sleep quality and over half reported threshold clinical insomnia, compared to the control group who reported 7% poor sleep quality and no clinical insomnia.

Actigraphic measurement showed no difference between the CLBP participants and the controls in total sleep time at seven hours per night, or in sleep onset latency taking an average of 10 minutes. These findings were consistent with previous studies investigating sleep quality in adolescents with chronic pain [30], but inconsistent with actigraphy studies of adults with chronic pain, who reported shorter overall sleep time, ranging from 4.9 to 5.9 hours [8,31]. However, similar to previous work, awakenings after sleep onset were more frequent in the CLBP participants than controls [8,31]. This is the first study to utilise a discrete CLBP population, and the findings suggest that although CLBP patients wake frequently during the night, they sleep for longer time periods than patients with other chronic musculoskeletal conditions and for the same duration as healthy age and gender matched individuals.; the reason for this requiring further investigation.

Subjectively the CLBP patients reported their sleep onset latency to be four times longer than actigraphy measures which is consistent with several studies that found sleep latency was one of the most frequent problems associated with chronic pain, with ranges from 20 to 102 minutes reported [1,2,7]. By interpreting most self report variables of sleep, the CLBP participants in this study had overall sleep quality that was comparable to other studies of patients with chronic pain [2,3,6-8,10,11].

Both self report and actigraphy revealed sleep efficiencies within the normal parameters (≥ 85%) for the control participants. However, the CLBP participants' sleep efficiency was well below the 85% criterion used to differentiate between good and bad sleepers [26]. Similarly, sleep efficiency values ranging from 75% to 87% have been found in other chronic pain studies [8,19,31,32]. The use of actigraphy in this study draws attention to the discrepancies that can occur with reliance on one type of measurement alone and emphasised the importance of including both subjective and objective measurement for complete, accurate sleep assessment.

In this study, the association between the subjective and objective measures varied significantly between both sleep variables groups. There was no association between the measures of sleep onset latency and sleep efficiency in the CLBP group, with self-report sleep onset latency being reported to a greater extent and sleep efficiency much lower. In contrast, control group self-reported sleep onset latency (r = .73) and sleep efficiency (r = .42) were strongly associated with actigraph measures. Discrepancies between self reporting and objective measurement of sleep have been previously reported [9,28,33,34] in several clinical populations. For example, adult cystic fibrosis patients reported higher sleep latency scores and lower sleep efficiency scores although actigraph measures revealed no differences [34]. Similarly, adults with nocturnal asthma reported reduced overall sleep but not increased night time awakenings even though polysomnographic assessment showed differences in sleep efficiency, latency and awakenings [33]. A study investigating a chronic pain population showed that the sleep diaries and actigraphs provided similar estimates of total sleep time, time awake after sleep onset and sleep efficiency, but differed in sleep latency and nocturnal awakenings [8].

Our results indicate that the subjective and objective findings differ to a great extent and that if sleep analysis was based exclusively on one measure over the other, the extent of the sleep disturbance in this population would be inaccurate. It therefore highlights the importance of using an objective assessment tool, such as the actigraph, as an adjunct to self report measures. Future studies of sleep quality and CLBP should be informed by this fact and should employ a detailed clinical interview and objective assessment to provide an accurate picture of overall sleep quality in this population.

The significance of the inconsistencies between the subjective and objective sleep variables in the CLBP group was especially obvious when investigating their association with pain, quality of life and disability. Only significant correlations between the clinical measures and sleep variables were observed with self report methods of assessment: the corresponding correlations with the actigraph derived variables were low and non-significant, again emphasising the importance of utilising both self-report and objective measurement to establish accurate relationships between sleep and other health variables.

As demonstrated, the subjective measures of sleep rather than objective are more consistent with pain, quality of life and disability measures and may incorporate negative subject bias and fears about sleep. Future research may establish differing clinical implications of poor sleep that is identified by subjective versus actigraphy measure, but preliminary investigation into the clinical correlates of sleep quality in CLBP participants from this study suggest that self report sleep quality is associated with pain and reduced physical function.

The strengths of this study include the use of both subjective and objective sleep measures and an age and gender matched healthy comparison group. Also, it is the first study that has investigated sleep disturbance both subjectively and objectively in one specific group of patients; chronic low back pain sufferers. However, as it was a pilot study, its principle limitation was that the sample size was small and therefore underpowered to identify smaller effects. Further investigation is now ongoing in a larger sample size over a longer period of time.

## Conclusion

The findings of this study serve to highlight the significance of the problem of sleep disturbance for CLBP patients and have important clinical implications for its assessment. The rate of self perceived poor sleep is very common in CLBP patients attending for clinical treatment, however, very little is understood about the relationship between CLBP and sleep disruption. Initial assessment of these patients should include sleep and where possible, a combination of both self-report and objective measures should be employed. Because the direction of causation between pain and sleep disturbance is still unclear and a recent perspectives indicate that poor sleep can interfere with pain processing [35], effective interventions to tackle sleep disturbance, such as cognitive behavioural therapy [36] should be included the overall management of the CLBP patient, to optimise treatment outcome.

## Competing interests

The authors declare that they have no competing interests.

## Authors' contributions

DAH and CH conceived the project. GO'D drafted the manuscript. All authors contributed to the study design, critically revised and approved the final manuscript.

## Pre-publication history

The pre-publication history for this paper can be accessed here:



## References

[B1] Rives PA, Douglass AB (2004). Evaluation and treatment of low back pain in family practice. Journal of American Board of Family Practice.

[B2] Marin R, Cyhan T, Miklos W (2006). Sleep Disturbance in Patients with Chronic Low Back Pain. American Journal of Physical Medicine & Rehabilitation.

[B3] Tang NKY, Wright KJ, Salkovskis PM (2007). Prevalence and correlates of clinical insomnia co-occuring with chronic back pain. J Sleep Res.

[B4] Marty M, Rozenberg S, Duplan B, Thomas P, Duquesnoy B, Allaert F (2008). Quality of sleep in patients with chronic low back pain: a case-control study. European Spine Journal.

[B5] Call-Schmidt TA, Richardson SJ (2003). Prevalence of Sleep Disturbance and its Relationship to Pain in Adults with Chronic Pain. Pain Management Nursing.

[B6] Morin CM, Gibson D, Wade J (1998). Self-Reported Sleep and Mood Disturbance in Chronic Pain Patients. Clinical Journal of Pain.

[B7] Pilowsky I, Crettenden I, Townley M (1985). Sleep disturbance in pain clinic patients. Pain.

[B8] Wilson KG, Watson ST, Currie SR (1998). Daily diary and ambulatory activity monitoring of sleep in patients with insomnia associated with chronic musculoskeletal pain. Pain.

[B9] Menefee LA, Frank ED, Doghramji K (2000). Self reported sleep quality for individuals with chronic pain. Clinical Journal of Pain.

[B10] Menefee LA, Cohen MJM, Anderson WR, Doghramji K, Frank ED, Lee H (2000). Sleep Disturbance and Nonmalignant Chronic Pain: A Comprehensive Review of the Literature. Pain Medicine.

[B11] Harman K, Pivik RT, D'Eon JL (2002). Sleep in depressed and non-depressed participants with chronic low back pain: electroencephalographic and behavioral findings. Sleep.

[B12] Sadeh A, Acebo C (2002). The role of actigraphy in sleep medicine. Sleep Medicine Reviews.

[B13] Vitello MV, Moe KE, Larsen LH, Prinz PN (1997). Age related sleep change: relationships of objective and subjective measures of sleep in healthy older men and women. Sleep Research.

[B14] Coates TJ, Killen J, George J, Marchini E, Hamilton S, Thoresen C (1982). Estimating sleep parameters: a multitrait-multimethod analysis. Journal of Clinical Psychology.

[B15] Buysee DJ, Reynolds CF, Monk TH, Berman SR, Kupfer DJ (1989). The Pittsburgh Sleep Quality Index: a new instrument for psychiatric practice and research. Psychiatry Research.

[B16] Batien CH, Vallie'res A, Morin CM (2001). Validation of the Insomnia Severity Index as an outcome for insomnia research. Sleep Med Rev.

[B17] Blake C, Codd MB, O'Mears YM (2000). The Short Form 36 (SF-36) Health Survey: a normative data for the Irish population. Irish Journal of Medical Science.

[B18] Fairbank J, Pynsent P (2000). The Oswestry Disability Index. Spine.

[B19] Haythornwaite JA, Hegel MT, Kerns RD (1991). Development of a sleep diary for chronic pain patients. Journal of Pain Symptom Management.

[B20] Cambridge Neurotechnology Ltd Actiwatch-Score Activity Monitoring System, User Manual. http://www.camntech.co.uk.

[B21] Smith MT, Wegener S (1993). Measures of sleep: The Insomnia Severity Index, Medical Outcomes Study (MOS) Sleep Scale, Pittsburgh Sleep Diary, and Pittsburgh Sleep Quality Index. Arthritis Care and Research.

[B22] Currie SR, Wilson KG, Curran D (2002). Clinical significance and predictors of treatment response to cognitive behavioral therapy for insomnia secondary to chronic pain. Journal of Behavioral Medicine.

[B23] Smith MT, Perlis ML, Smith MS, Giles DE (2000). Sleep quality and pre-sleep arousal in chronic pain. Journal of Behavioral Medicine.

[B24] Smith S, Trinder S (2001). Detecting insomnia: comparison of four self-report measures of sleep in a young adult population. Journal of Sleep Research.

[B25] Monk TH, Reynolds CF, Kupfer DJ, Buysse DJ, Coble PA, Hayes AJ (1994). The Pittsburgh Sleep Diary. Journal of Sleep Research.

[B26] Morin CM (1993). Insomnia: psychological assessment and management.

[B27] Anderson GB (1999). Epidemiological features of Chronic Low Back Pain. Lancet.

[B28] Sayer K, Arikan M, Yontem T (2002). Sleep quality in chronic pain patients. Canadian Journal of Psychiatry.

[B29] American Sleep Disorders Association (1995). Practice Parameters for the use of actigraphy in the clinical assessment of sleep disorders. Sleep.

[B30] Palermo TM, Toliver-Sokol M, Fonareva I, Koh JL (2007). Objective and Subjective Assessment of Sleep in Adolescents with Chronic Pain Compared to Healthy Adolescents. Clinical Journal of Pain.

[B31] Lavie P, Epstein R, Tzischinsky D, Gilad D, Nahir M, Lorber M, Scharf Y (1992). Actigraphic measurements of sleep in rheumatoid arthritis: comparison of patients with low back pain and healthy controls. Journal of Rheumatology.

[B32] Wittig RM, Zorick FJ, Blumer D, Heilbrom M, Roth T (1982). Disturbed sleep in patients complaining of chronic pain. Journal of Nervous Mental Disorders.

[B33] Fitzpatrick MF, Engleman H, Whyte KF, Deary IJ, Shapiro CM, Douglas NJ (1991). Morbidity in nocturnal asthma: sleep quality and daytime cognitive performance. Thorax.

[B34] Jankelowitz L, Reid KJ, Wolfe L, Cullina J, Zee PC, Jain M (2005). Cystic Fibrosis Patients Have Poor Sleep Quality Despite Normal Sleep Latency and Efficiency. Chest.

[B35] Lautenbacher Lautenbacher S, Kundermann B, Krieg J (2006). Sleep Deprivation and Pain Perception. Sleep Medicine Reviews.

[B36] Smith MT, Haythorntwaithe JA (2004). How do sleep disturbance and chronic pain inter-relate? Insights from the longitudinal and cognitive-behavioural clinical trials literature. Sleep Medicine Reviews.

